# Contrasting arbuscular mycorrhizal communities colonizing different host plants show a similar response to a soil phosphorus concentration gradient

**DOI:** 10.1111/nph.12169

**Published:** 2013-02-20

**Authors:** Paul Gosling, Andrew Mead, Maude Proctor, John P Hammond, Gary D Bending

**Affiliations:** 1 HGCAStoneleigh Park, Kenilworth, Warwickshire, CV8 2TL, UK; 2 School of Life Sciences, University of WarwickCoventry, CV4 7AL, UK; 3 National Pollen and Aerobiology Research Unit, Charles Darwin Building, University of WorcesterHenwick Grove, Worcester, WR2 6AJ, UK; 4 School of Plant Biology, The University of Western AustraliaCrawley, WA, 6009, Australia

**Keywords:** colonization, diversity, maize (*Zea mays*), mycorrhizas, phosphorus (P), soybean (*Glycene max*), *Viola arvensis* (field violet)

## Abstract

- High soil phosphorus (P) concentration is frequently shown to reduce root colonization by arbuscular mycorrhizal (AM) fungi, but the influence of P on the diversity of colonizing AM fungi is uncertain.
- We used terminal restriction fragment length polymorphism **(T-RFLP)** of 18S rDNA and cloning to assess diversity of AM fungi colonizing maize (*Zea mays*), soybean (*Glycene max*) and field violet (*Viola arvensis*) at three time points in one season along a P gradient of 10–280 mg l^−1^ in the field.
- Percentage AM colonization changed between sampling time points but was not reduced by high soil P except in maize. There was no significant difference in AM diversity between sampling time points. Diversity was reduced at concentrations of P > 25 mg l^−1^, particularly in maize and soybean. Both cloning and T-RFLP indicated differences between AM communities in the different host species. Host species was more important than soil P in determining the AM community, except at the highest P concentration.
- Our results show that the impact of soil P on the diversity of AM fungi colonizing plants was broadly similar, despite the fact that different plants contained different communities. However, subtle differences in the response of the AM community in each host were evident.

## Introduction

Arbuscular mycorrhizal (AM) fungi are an important component of the soil microbial community. They form a symbiotic relationship with *c*. 90% of terrestrial plant species, supplying nutrients, particularly phosphorus (P), to the host plant in exchange for carbon (Smith & Read, 2008). This symbiotic relationship can also result in increased resistance to drought, soilborne fungal pathogens and heavy metals in the host plant. There is also evidence to indicate that AM fungi improve soil structure (Rillig & Mummey, 2006).

The diversity of AM fungi is low relative to that of host plants, with *c*. 230 species described morphologically (http://schuessler.userweb.mwn.de/amphylo/), compared with the estimated 300 000 terrestrial plant species (Mora *et al*., 2011), although evidence from molecular methods suggests that the true diversity of AM fungi is somewhat higher (Opik *et al*., 2009; Lumini *et al*., 2010). Despite the relatively low diversity of AM fungi, there are evident differences between AM communities in different habitats and between AM fungi colonizing different host species in the same habitat (Helgason *et al*., 1998; Scheublin *et al*., 2004; Uibopuu *et al*., 2009; Hazard *et al*., 2012). The mechanisms driving differences in AM fungi colonizing different host species are unclear, but seem likely to be attributable, at least in part, to functional differences between AM fungi (Maherali & Klironomos, 2007). Distinctions between AM fungal communities at different sites or in different habitats may be partially explained by variation in host plant occurrence, but are also a result of environmental factors. For instance, agricultural practices have been repeatedly shown to impact AM symbioses (Gosling *et al*., 2006), with more intensive production systems having both reduced AM populations and AM diversity when compared with more extensive or pastoral production systems (Hijri *et al*., 2006; Gosling *et al*., 2010; Van der Gast *et al*., 2011).

One environmental factor shown to have a negative impact on the AM symbiosis is a high concentration of extractable soil P. Assimilation of P by AM colonized plants reflects the sum of uptake directly via plant cells, and indirectly via AM fungi, with the importance of the AM pathway declining when P availability is high, which is usually associated with a decline in AM colonization (Smith & Smith, 2011). These effects are mediated via the impact on host nutrition and, although the exact mechanisms involved are uncertain, changes in expression of host genes related to the different pathways of P uptake, with consequent changes to host signalling responsible for regulating formation of the symbiosis, are likely to be involved (Balzergue *et al*., 2011; Smith & Smith, 2011).

Recently, using *in vitro* tissue culture systems, Kiers *et al*. (2011) suggested that the trade in host C for fungal P could control the precise fungal communities colonizing roots, the plant selecting fungi which provide P at the lowest C cost by preferentially allocating them C. In turn, AM fungi preferentially transfer P to plants with the highest C supply. The extent to which the plant specifically rewards other benefits provided by individual AM species, such as uptake of N and water, and pathogen suppression is unknown.

The way in which the interplay of trade in fungal P for host C is impacted by P availability is uncertain. While colonization of host roots by AM fungi and the AM P uptake pathway is suppressed and may even be eliminated at high soil P concentrations (Jensen & Jakobsen, 1980; Hicks & Loynachan, 1987; Thingstrup *et al*., 1998; Ryan & Ash, 1999; Khaliq & Sanders, 2000; Liu *et al*., 2000; Kahiluoto *et al*., 2001), suggesting reduced C allocation to AM fungi, the relationship between soil P and AM diversity is unclear.

Plant species have varying requirements for P, reflecting differences in biomass and cellular concentration, and this requirement can change between developmental stages (Raghothama, 1999). The value of AM-derived P, in terms of its C cost, is therefore likely to vary between plant species and with developmental stage. It is also likely to depend on the supply of P available via direct and mycorrhizal pathways.

Counts of AM spores in the soil suggest that high concentrations of available soil P can reduce diversity, or at least populations of some species of AM fungi in the soil (Johnson, 1993; Hamel *et al*., 1994; Kurle & Pfleger, 1996), but results based on counts of AM spores in the soil are unlikely to reflect activity within host roots. Hijri *et al*. (2006) suggested that lower diversity of AM fungi colonizing roots in some agricultural fields compared with others was attributable to soil P, but there were significant confounding influences, not the least of which were differences in the host plants examined.

We therefore sought to assess the following. Does a high concentration of available soil P have a negative impact on colonization of a range of host plants by AM fungi? Does a high concentration of available soil P alter the diversity of AM fungi colonizing a range of host plants? Does the effect of soil P depend on host identity? Is there is an interaction between induced shifts in AM community diversity and growth stage?

## Materials and Methods

### Experimental site

**Figure 1:**
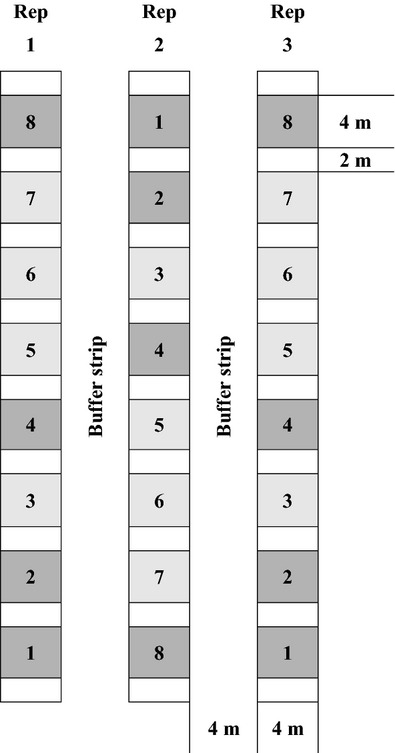
Diagram of one set of blocks in field experimental design (identical for each species). Numbers in trial plots indicate target soil phosphorus (P) concentration (Table 1). Sampled plots are shown in dark grey, buffer areas are unshaded.

We utilized established historical soil P gradients within an agricultural field on the Wellesbourne Campus of the University of Warwick, UK (52º12′34″N, 1º36′37″W) for these experiments. Mean annual temperature at the site is 10.4°C and mean annual rainfall is 598 mm. The soil is an inceptisol (USDA Soil Taxonomy) of the Wick series with a sandy loam texture (Whitfield, 1974). Soil P concentration gradients were established in 2002 to measure the response of various brassica crops to soil P concentrations (Fig. 1). The field experiment was arranged in three sets of three replicate blocks, each block containing a systematic arrangement of eight soil P concentrations. The systematic arrangement was used to minimize the need for guard areas between plots with very different soil P concentrations (Mead *et al*., 2012), with the direction of the systematic trend being alternated between adjacent blocks. This allowed a *post hoc* adjustment for distance from the field edge across the blocks to be included in the analyses, along with an allowance for variation in the response between blocks, providing adjustment in the responses for any consistent pattern of spatial variability.

After 2 yr of growing brassicas, the plots were subsequently used for potatoes and wheat, with the soil P gradients maintained by annual measurement and adjustment of P concentrations through P fertilizer additions, allowing time for AM communities to respond to the new soil P concentrations. In 2007, the gradients were used to measure the effect of soil P on three crop species: spring oilseed rape (OSR) (*Brassica napus* L.) cv ‘Senator’, soybean (*Glycene max* L.) cv ‘Elena’ and forage maize (*Zea maize* L.) cv ‘PR39 G12’. The soil was spaded, tinned and power-harrowed before planting. Phosphorus was applied by hand as triple superphosphate sufficient to maintain eight soil P concentrations equivalent to the UK agricultural soil P index system, index values 1–8 (Table 1). P application to the soil had no detectable impact on soil pH, which had remained at *c*. 7.2 since 2002. OSR was sown on 18 April, and maize and soybean were sown on 6 June.

**Table 1:** Target soil phosphorus (P) concentrations, based on the Olsen extraction procedure, as described in MAFF (1986), *The Analysis of Agricultural Materials*, and Resin P (Hislop & Cooke, 1968)

Soil P index*	Olsen’s P concentration (mg l^−1^)	Resin P (mg l^−1^)
1	10–15	20–30
2	16–25	31–49
3	26–45	50–85
4	46–70	86–132
5	71–100	> 132
6	101–140	
7	141–200	
8	201–280	

* Based on UK fertilizer recommendations – RB209 (MAFF, 2000) concentration.

### Plant sampling

Three plants were selected at random from guard rows in maize (plots consisted of two inner and two outer guard rows) and from three outer rows of the multiple rows in soybean, in the P1, P2, P4 and P8 plots. Plants were dug up using a garden fork and roots removed down to 30 cm. Excess soil was removed in the field and then the roots were carefully washed with deionized water to remove soil and blotted dry. Roots were chopped into 0.5 cm lengths and mixed. Approximately 500 mg of each sample was frozen at −20°C for molecular analysis and the remainder was used for assessment of colonization. As OSR is nonmycorrhizal, the OSR gradient was used to test AM fungal colonization and diversity in a weed species. Weeds may play an important role in agroecosystems by maintaining AM fungal populations during periods between crops or during the cultivation of nonmycorrhizal crops such as brassicas (Kabir & Koide, 2000). No weed control was used on OSR plots, allowing a diverse weed flora to develop. We assessed AM colonization and diversity in field violet (*Viola arvensis* L.) on these plots. It is a relatively common weed at this site, with a strong competitive ability (Storkey, 2006). Preliminary examination showed that it was frequently colonized by AM fungi and it was common in the OSR plots. However, its distribution was patchy and it became apparent that it was not sufficiently common in one of the OSR blocks to sample all plots and so only two replicate blocks were sampled for *Viola*. Three *Viola* plants were selected at random from across each sampled plot at the appropriate growth stage as described in the following.

Plants were sampled at three distinct developmental stages of the crop species: preflowering; immediately after flowering; and at seed maturity. The first sampling was conducted on 24 July. The second sampling was done on the 6 September with the third sampling on the 4 October – (for soybean and *Viola*) and the 25 October for maize. *Viola* has an indeterminate growth pattern and so plant selection for sampling was done as follows: first sampling, small young plants that had not initiated flowering; second sampling, large mature *Viola* plants flowering profusely; third sampling, plants with seed set and few remaining flowers.

### AM colonization

Arbuscular mycorrhizal colonization was measured on roots from each replicate stained according to the method of Grace & Stribley (1991). Root colonization was quantified using the gridline intersect method, and 100 intersects were assessed for each sample.

### AM diversity

DNA was extracted from 50 mg FW root samples using a Biogene soil DNA kit following the manufacturer’s instructions. AM fungal 18S rDNA (*c*. 800 bp) was amplified using the AM specific primers AML1 (ATCAACTTTCGATGGTAGGATAGA) and AML2 (GAACCCAAACACTTTGGTTTCC) described by Lee *et al*. (2008) labelled with the fluorescent dyes Hex and 6-FAM, respectively. The reaction mixture was as follows: 4 μl DNA (5–25 nmol μl^−1^), 1 μl of each primer (25 pm concentration) and 44 μl of MegaMix (Microzone Ltd, Haywards Heath, UK). PCR conditions were as follows: 3 min at 94°C followed by 40 cycles of 1 min at 94°C, 1 min at 57°C and 1 min at 72°C with a final extension of 5 min at 72°C. PCR products were cleaned using a QIAquick PCR purification kit (Qiagen).

In order to determine the most efficient restriction enzymes to use for terminal restriction fragment length polymorphism **(**T-RFLP) and to confirm the efficiency of the PCR reaction in eliminating nontarget organisms, two clone libraries were constructed, one each using PCR product from maize and soybean. The maize sample was from replicate 1, at the second harvest, in the lowest soil P concentration and the soybean sample from replicate 2 at the second harvest, in the lowest soil P concentration. The samples selected for cloning had high degrees of colonization. DNA was amplified as desribed earlier but using unlabelled primers and with a final extension time of 10 min. PCR product was cloned with a PCR cloning kit (Qiagen). Sequencing reactions were conducted using a PRISM BigDye Terminator Cycle Sequence reaction kit (Applied Biosystems, Foster City, CA, USA), with products analysed on an Applied Biosystems 377 automated DNA sequencer. DNA sequences were edited and assembled using the DNAstar II sequence analysis package (Lasergene Inc., Madison, WI, USA). Sequences were compared with those on the European Molecular Biology Laboratory (EMBL) DNA database by BLAST analysis.

Restriction enzymes were selected based on the resulting clone libraries using the Restriction Enzyme Mapping Application (REMA, http://bioperl.macaulay.ac.uk/) to give the maximum number of unique terminal restriction fragments (T-RFs) with a size > 75 bp (in order to avoid problems with primer peak in T-RFLP traces). The restriction endonucleases HpyCH4III and AseI were selected (Promega). Restriction digests were carried out separately for each enzyme. Samples were incubated at 37°C for 16 h and denatured for 15 min at 95°C. Digestion products were purified and run on an Applied Biosystems 377 automated DNA sequencer. T-RFLP peaks were determined with the aid of the GeneMarker computer programme (SoftGenetics LLC, State College, PA, USA).

### Statistics

Differences in colonization and mean number of T-RFLP peaks between plant species (all primer/enzyme combinations), P concentrations and sampling points were analysed using ANOVA. Distance across the field was initially included in the models as a covariate to account for the nonrandom plot positioning, but proved not to be significant. Therefore it was removed and data reanalysed. Data were log-transformed as necessary to meet assumptions of ANOVA. Differences between means where ANOVA was significant were assessed using Tukey’s honestly significant difference (HSD) test.

The AM fungal communities colonizing plant roots were compared by nonmetric multidimensional scaling (NMDS) using presence/absence data, with dissimilarities calculated using the zero-adjusted Bray–Curtis method (Clarke *et al*., 2006). NMDS is an iterative ordination method that is highly effective at revealing relationships in ecological community data (McCune & Grace, 2002). Its advantage is that it makes very few assumptions about the relationship between the variable gradient and the pattern of response of the community. In addition it can be used with any dissimilarity measure and it has seen increasingly widespread use in the last 10 yr (von Wehrden *et al*., 2009). The significance of differences between treatments after NMDS was assessed using pairwise comparison of all pairs of treatments with a multiple response permutation procedure (MRPP). Significance levels were not adjusted for the effect of multiple comparisons, but where such an adjustment based on the Šidák correction (Abdi, 2007) would alter the result, this is reported in the text. To determine which T-RFs were contributing most to differences identified by MRPP, similarity percentage analyses (SIMPER) were performed. This method compares average abundances and examines the contribution of each T-RF to similarities within a given group or dissimilarities between groups (Clarke & Warwick, 2001).

To determine the relative importance of soil P and host species in determining the structure of the AM community, the total number of individual T-RF occurrences across the three sampling points was determined for each host species at each soil P concentration. Resemblance of each pair of T-RF communities was determined using the zero-adjusted Bray–Curtis method and these were used to perform a hierarchical agglomerative cluster analysis using group average clustering. The significance of group linkage was tested using a permutation test.

Phylogenic analysis of cloned sequences was performed in Phylip 3.68, ANOVAs were calculated in GenStat version 13.3, SIMPER analyses and cluster analyses were performed using PRIMER version 6 (Clarke & Warwick, 2001), and all other analyses were performed using PC-Ord version 5.06 (McCune & Mefford, 2006).

## Results

### AM colonization is affected by soil P concentration and sampling time

Root colonization by AM fungi was generally low to moderate across all species, with few samples above 30% colonization and a substantial number below 10% at the first and last sampling time points (Table 2). Colonization was particularly low in soybean at the first harvest, no sample exceeding 8% colonization. Colonization was highest in maize (1–64%), the mean of which was significantly (*P* < 0.05) greater than in soybean (1–50%) and *Viola* (0–27%) across all soil P concentrations, with the highest degree of colonization in an individual plot sample being in maize at all three sampling times (H1, 23%; H2, 64%; H3, 52%; Table 2). Sampling time had a significant (*P* < 0.05) influence on colonization in all three plant species. Mean percentage colonization more than doubled between the first and second sampling times in maize and *Viola* at all soil P concentrations, with a large increase also recorded in soybean. Colonization then declined between sampling times 2 and 3 in all plant species, although this decline was only significant for soybean (from 17.5 to 6.8% across all P concentrations). In maize, percentage root colonization by AM fungi was significantly reduced at the two highest soil P concentrations, from 29.2% across all sampling times at P1 to 13.7% at P4 and 10.6% at P8, but soil P concentration had no significant effect on percentage colonization of soybean or *Viola* and there was little evidence of a trend in the data.

**Table 2:** Mean percentage root length colonized by arbuscular mycorrhizal (AM) fungi in maize, soybean and *Viola* at four concentrations of soil phosphorus (P) and three sampling times, with significance level for ANOVA, standard errors given in italics and means that differ significantly indicated by different lower-case letters (Tukey’s honestly significant difference (HSD) test)

	Maize				Soybean				*Viola*			
	Sampling time point				Sampling time point				Sampling time point			
Soil P concentration	1	2	3	Overall mean	1	2	3	Overall mean	1	2	3	Overall mean
P1	10.7	44.7	32.3	29.2 a	6.7	12.0	9.0	9.2	10.0	25.0	14.5	16.5
	*4.8*	*11.9*	*3.2*	*6.3*	*0.7*	*5.5*	*3.6*	*2.1*	*6.0*	*2.0*	*7.5*	*3.8*
P2	14.7	31.0	26.7	24.1 ab	3.0	35.7	6.3	15.0	9.0	21.5	14.0	14.8
	*4.3*	*7.1*	*12.7*	*5.0*	*0.6*	*7.4*	*0.9*	*5.6*	*7.0*	*2.5*	*2.0*	*3.0*
P4	3.3	21.0	16.7	13.7 bc	5.7	10.3	6.0	7.3	4.5	18.5	22.0	15.0
	*1.9*	*7.6*	*2.9*	*3.6*	*1.5*	*3.2*	*1.5*	*1.3*	*1.5*	*3.5*	*5.0*	*3.8*
P8	3.0	12.0	16.7	10.6 c	2.7	12.0	6.0	6.9	6.0	21.5	6.5	11.3
	*1.5*	*1.0*	*3.2*	*2.3*	*1.2*	*6.7*	*6.0*	*2.7*	*6.0*	*0.5*	*1.5*	*3.6*
Overall mean across P concentrations	7.9 a	27.2 b	23.1 b		4.5 a	17.5 b	6.8 a		7.4 a	21.6 b	14.2 ab	
	*2.1*	*5.0*	*3.6*		*0.7*	*4.1*	*1.3*		*2.3*	*1.3*	*2.7*	
Phosphorus effect	*P* = 0.002				*P* = 0.076				*P* = 0.564			
Sampling time effect	*P* < 0.001				*P* < 0.001				*P* = 0.003			
Interaction	*P* = 0.503				*P* = 0.014				*P* = 0.484			

### Molecular analysis

A total of 87 clones were sequenced from maize and 78 from soybean. Representative sequences were deposited in the EMBL Nucleotide Sequence Database, accession numbers FR848583–FR848644 inclusive. There were no contaminant sequences in either maize or soybean clone libraries. Phylogenetic analysis of nonidentical clones along with sequences of described AM fungal species produced a topology in close agreement with that published by Schwarzott *et al*. (2001) and others published in more recent work (e.g. Santos *et al*., 2006; Opik *et al*., 2009; Supporting Information Fig. S1). Seventy-five per cent of the sequences isolated from maize clustered with described sequences in the putative family *Glomus* group A, (Schwarzott *et al*., 2001) with 25% clustering with *Glomus* group B, while 95% of sequences isolated from soybean clustered with *Glomus* group A and 5% with *Glomus* group C (Diversisporaceae). Unfortunately, available restriction enzymes provide poor discrimination between Glomeraceae in the 18S region, which, combined with the small inherent uncertainty in T-RF sizing associated with T-RFLP, makes it impossible to directly associate T-RFs with named sequences.

### AM diversity varies between plant species and with P concentration

The total number of T-RFs (both labelled fragments and both restriction enzymes) found in soybean (85) and *Viola* (84) were very similar and considerably more than in maize (55). However, the mean number of T-RFs across all P concentrations and sample times was highest in soybean (24.6), with maize (15.1) and *Viola* (18.9) not differing significantly (*P* < 0.001). In maize, soil P concentration had a significant (*P* < 0.001) influence on the total number of T-RFs (Table 3). P1 (22.1) had significantly more T-RFs on average than both P4 (8.6) and P8 (11.1), while P2 (18.7) had significantly more peaks than P4 but not P8. There was no significant effect of sampling time on the number of T-RFs in maize. In soybean, the number of peaks in P8 (9.6) was significantly lower than all other soil P concentrations, with fewer than half the number at P8 than at any other soil P concentration at all sampling times. Sampling time had no significant effect on the total number of T-RFs in soybean. In *Viola* neither sampling time nor soil P concentration had a significant effect on the total number of T-RFs, although there was a definite trend towards lower numbers of T-RFs at P8 in *Viola*, with 9.8 compared with 20.3–23.5 in P1–P4, and the *P* value was somewhat marginal (Table 3).

**Table 3:** Mean number of terminal restriction fragments (T-RF) for the three plant species over three sampling times at four concentrations of soil phosphorus (P), with significance level for ANOVA, standard errors given in italics and means that differ significantly indicated by different lower-case letters (Tukey’s honestly significant difference (HSD) test)

	Maize				Soybean				*Viola*			
	Sampling time point				Sampling time point				Sampling time point			
Soil P concentration	1	2	3	Overall mean	1	2	3	Overall mean	1	2	3	Overall mean
P1	17.3	23.3	25.7	22.1 a	35.7	32.3	33.0	33.7 a	16.5	18.5	26.0	20.3 a
	*4.7*	*1.8*	*4.2*	*2.3*	*1.5*	*1.6*	*5.6*	*1.8*	*9.5*	*0.5*	*2.0*	*3.1*
P2	16.7	17.0	22.3	18.7 ac	29.7	27.7	23.3	26.9 a	34.0	16.5	20.0	23.5 a
	*5.2*	*1.5*	*0.9*	*1.8*	*0.3*	*6.7*	*2.2*	*2.2*	*9.0*	*0.5*	*11.0*	*5.0*
P4	5.3	9.3	11.0	8.6 bd	31.7	30.7	22.7	28.3 a	24.0	16.5	25.0	21.8 a
	*1.2*	*1.2*	*2.5*	*1.2*	*3.5*	*2.7*	*5.2*	*2.4*	*1.0*	*1.5*	*11.0*	*3.3*
P8	8.7	11.3	13.3	11.1 cd	13.7	6.7	8.3	9.6 b	5.5	8.0	16.0	9.8 a
	*5.5*	*5.7*	*6.7*	*3.1*	*5.3*	*3.5*	*4.1*	*2.4*	*4.5*	*8.0*	*3.0*	*3.9*
Overall Mean across P concentrations	12.0	15.2	18.1		27.7	24.3	21.8		20.0	14.9	21.8	
	*2.5*	*2.1*	*2.6*		*2.9*	*3.6*	*3.3*		*4.7*	*2.2*	*3.4*	
Phosphorus effect	*P* < 0.001				*P* < 0.001				*P* = 0.078			
Sampling time effect	*P* = 0.104				*P* = 0.072				*P* = 0.303			
Interaction effect	*P* = 0.992				*P* = 0.808				*P* = 0.487			

**Figure 2:**
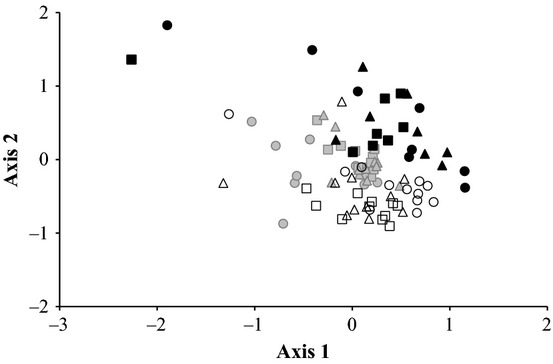
Nonmetric multidimensional scaling ordination plot showing terminal restriction fragment (T-RF) communities in maize (grey symbols), soybean (white symbols) and *Viola* (black symbols) at first (circles), second (squares) and third (triangles) sampling time points. Results of 500 iterations, 250 each with real and randomized data (Monte Carlo test). Final stress for two dimensions, 17.44; final instability, 0.0022. Variation in distance matrix represented by: axis 1, 56.3%; axis 2, 22.9%.

Nonmetric multidimensional scaling of TR-F data revealed that there were large differences in the makeup of the T-RF communities in each plant species. Across all soil P concentrations and sampling times, there was a significant difference in the clustering of the three plant species (MRPP, *P* < 0.001; Fig. 2). Maize and soybean showed relatively little dispersion within the dataset, along either axis 1 or 2, while *Viola* showed considerably more dispersion, particularly along axis 1. Pairwise comparisons indicated that differences between T-RF communities in the different plant species were significant an all cases (*P* < 0.001). Maize and soybean T-RF communities clustered closer together (Fig. 2), suggesting these were more similar to each other than they were to the *Viola* T-RF community. Examination of T-RF frequencies revealed that, of the five most common T-RFs, present in 75% or more of maize samples, four were also present in 75% or more of soybean samples and the fifth was present in 73% or more of soybean samples. By contrast, of the five most common T-RFs in *Viola*, only one was found at a frequency of 75% or more in soybean or maize samples.

There were some T-RFs found exclusively in soybean or *Viola*, including two in *Viola* that occurred in > 50% of samples and one in soybean that occurred in 25% of samples. By contrast, there were no T-RFs found exclusively in maize.

**Figure 3:**
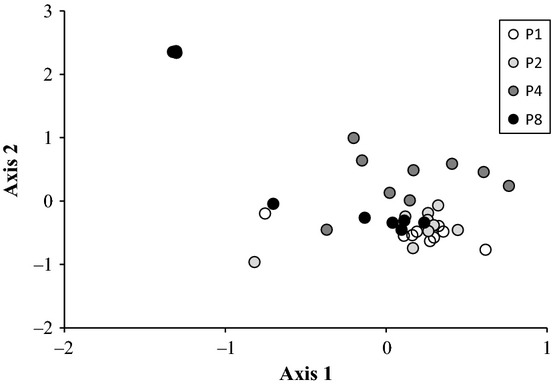
Nonmetric multidimensional scaling ordination plot showing terminal restriction fragment (T-RF) communities in maize at different soil phosphorus (P) index values: P1, P2, P4 and P8. Results of 500 iterations, 250 each with real and randomized data (Monte Carlo test). Final stress for two dimensions, 11.87; final instability, 0.0003. variation in distance matrix represented by: axis 1, 65.5%; axis 2, 21.1%.

Considering maize alone, there was no significant effect of sampling time on the composition of AM communities (*P* = 0.29, data not shown). This mirrors the results for the ANOVA of total T-RF number (Table 3). The effect of P concentration on the composition of the AM community in maize was significant (Fig. 3), (*P* < 0.001). The TR-F community in P4 samples was significantly different from that in P1 and P2 (*P* < 0.001) and from that in P8 (*P* = 0.014) (although adjusting for multiple comparisons would make the difference from P8 nonsignificant). The AM community in P8 was significantly different from those in P1 and P2 samples, *P* = 0.015, and *P* = 0.038, respectively (though these differences are not significant if accounting for multiple comparisons). Again, these results closely mirror the results of the ANOVA of total peak number (Table 3). SIMPER analysis did not identify any particular T-RFs as having a disproportionately large influence on the difference between P concentrations. Direct examination of T-RFs showed, instead, that there were a large number of T-RFs found in P1 and P2 at all harvests that were absent in P4. The situation was similar when comparing P1 and P2 with P8, with several T-RFs found in most or all replicates at the lower P concentrations, but only in two or three replicates at P8.

**Figure 4:**
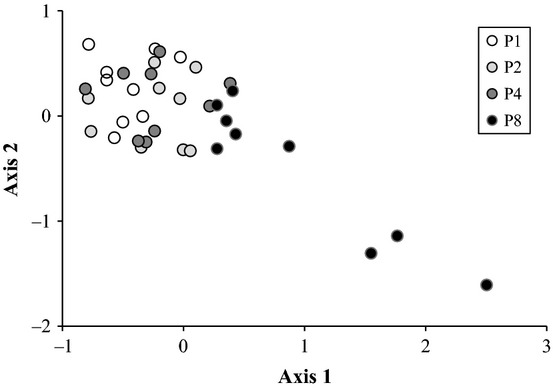
Nonmetric multidimensional scaling ordination plot showing terminal restriction fragment (T-RF) communities in soybean at different soil phosphorus (P) index values: P1, P2, P4 and P8. Results of 500 iterations, 250 each with real and randomized data (Monte Carlo test). Final stress for two dimensions, 14.06; final instability, 0.00056. variation in distance matrix represented by: axis 1, 75.5%; axis 2, 6.4%.

In soybean, sampling time had no significant effect on T-RF community composition, (*P* = 0.089, data not shown). The effect of P concentration on the composition of the T-RF community was significant (*P* < 0.001). The NMDS analysis suggests that P8 samples were very different from all other P concentrations, with very little overlap with other samples along axis 1 (Fig. 4). Pairwise MRPP comparisons confirmed significant differences between P8 and all other P concentrations (*P* < 0.001 in all cases), but no significant differences between any other P concentrations. This mirrors the analysis of numbers of T-RFs, which were also significantly lower in P8 than in other P concentrations, with no other P concentrations differing significantly (Table 3). SIMPER analysis indicated that there were no T-RFs that contributed disproportionately to the difference between P8 and the other P concentrations, rather there was a general reduction in the number of T-RFs.

**Figure 5:**
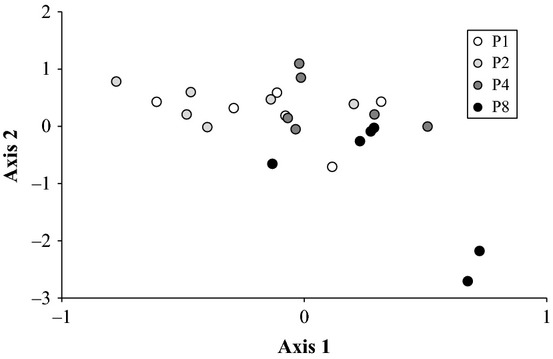
Nonmetric multidimensional scaling ordination plot showing terminal restriction fragment (T-RF) communities in *Viola* at different soil phosphorus (P) index values: P1, P2, P4 and P8. Results of 500 iterations, 250 each with real and randomized data (Monte Carlo test). Final stress for two dimensions, 17.86; final instability, 0.0013. variation in distance matrix represented by: axis 1, 32.2%; axis 2, 36.2%.

In *Viola*, there was no significant difference between the T-RF communities at different sampling times (*P* = 0.81, data not shown). By contrast, P concentration had a significant influence on the composition of the T-RF communities (*P* = 0.002), in contrast to the result from the ANOVA of T-RF peak numbers (Table 3). Again P8 samples were separated from the other P concentrations in the NMDS ordination (Fig. 5). Pairwise comparison between P concentrations revealed that differences between P1, P2, P4 and P8 were indeed significant (*P* = 0.013, *P* < 0.001 and *P* = 0.006, respectively; although the difference between P1 and P8 would not be significant if accounting for multiple comparisons). SIMPER analysis did not indicate that any T-RFs contributed disproportionately to the difference between P8 and the other P concentrations; rather, as with maize and soybean, there was a general reduction in the frequency of many T-RF’s.

### Relative importance of host plant identity and soil P

**Figure 6:**
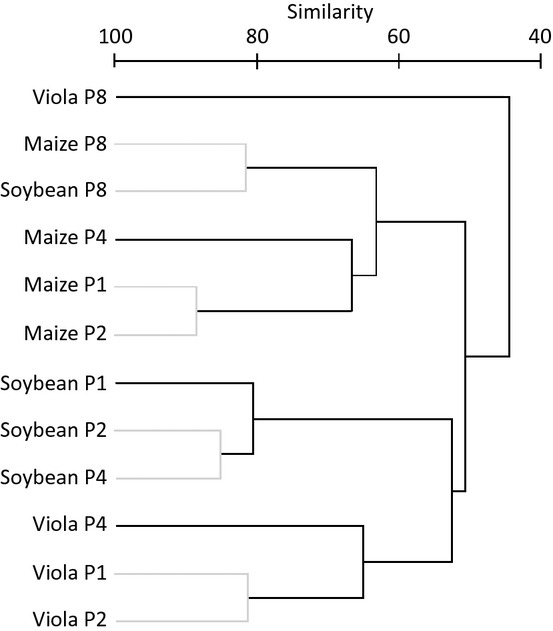
Hierarchical cluster analysis of terminal restriction fragment (T-RF) communities based on zero-adjusted Bray–Curtis similarity. Samples connected by grey lines cannot be significantly differentiated at *P* < 0.05 (Permutation test).

Soil P concentration and host plant species both had a significant impact on the AM community colonizing roots of the three plants. The relative importance of soil P and host plant species was observed through hierarchical cluster analysis of T-RF communities (Fig. 6). All three sampling times were combined, so this result reflects only the effect of soil P and host species. It is clear from this analysis that host plant species was more important than soil P concentration in determining the T-RF community. The T-RF communities in each host were more similar to each other at P1, P2 and P4 than to the T-RF community in the other host species at any soil P concentration. It was only at P8 that P concentration became more important at driving the AM community composition than host identity. The T-RF communities in maize and soybean at P8 were more similar to each other than to their respective communities at other soil P concentrations, while the T-RF community at P8 in *Viola* was highly dissimilar to all other T-RF communities.

## Discussion

The relationship between AM fungi and their plant host is strongly controlled by the host P status, which is in turn strongly influenced by soil P availability (Smith & Read, 2008). There is considerable evidence in the literature to support this hypothesis. A reduction in the percentage AM colonization of roots as extractable soil P concentration increases is often evident (Jensen & Jakobsen, 1980; Ryan *et al*., 1994; Kahiluoto *et al*., 2001), but the influence of soil P on the diversity of AM fungi colonizing roots is not so clear. Although some authors have suggested a link between reduced AM fungal diversity and high soil P (Hijri *et al*., 2006), others have suggested there is no connection (Mathimaran *et al*., 2005; Beauregard *et al*., 2010). Furthermore, published assessments of the effect of soil P on AM diversity are sometimes confounded by factors such as host species, and the true influence of soil P is uncertain.

Our results showed a complexity of response that may go some way to explaining the contradictory results in the literature. Although there was a clear response to soil P, it was only evident at higher soil P concentrations and was strongly mediated by host plant identity. While we did record a significant decrease in AM colonization in response to increasing soil P in maize, there was no significant decrease, even at the highest soil P concentrations, in soybean and *Viola*. The soil P concentration range used was very large and thus ought to have been sufficient to produce a response in all species, yet it did not. The lack of response in soybean was particularly unexpected, as large reductions in AM colonization of soybean have been reported in response to P fertilizer application (Hicks & Loynachan, 1987; Isobe *et al*., 2008), although Fernandez *et al*. (2011) reported a response of colonization in soybean to soil P only at low soil P concentrations, with no response above *c*. 15 mg kg^−1^ P (Bray 1 extraction). The fact that there was no response in *Viola* and soybean combined with the generally low to moderate degree of colonization in all three host species might suggest that the AM community at this site is degraded and dominated by AM fungal species with weak colonization potential and weak response to soil P. However, the degree of colonization we recorded was within the range reported in the literature for maize and soybean (Galvez *et al*., 2001; Ryan & Graham, 2002; Liu *et al*., 2003; Murillo-Williams & Pedersen, 2008; Duan *et al*., 2010; Fernandez *et al*., 2011) and the AM diversity analysis does not support this hypothesis.

Examination of clones did not indicate amplification of nontarget DNA (see Notes S1) and the mean number of T-RFs found was similar to that reported elsewhere for AM fungi in arable agricultural systems (Vandenkoornhuyse *et al*., 2003; Mummey & Rillig, 2008; Bainard *et al*., 2012), indicating that the background AM community diversity at this site was not unusual. The dominance of the AM community by *Glomus* spp., evident from the cloned sequences, is also common for tilled agricultural soils (Johnson *et al*., 1991; Hendrix *et al*., 1995; Kurle & Pfleger, 1996; Daniell *et al*., 2001; Oehl *et al*., 2003; Toljander *et al*., 2008), also suggesting there was nothing unusual about this site.

More significantly, AM diversity was significantly impacted by soil P in maize and soybean. The effect at P8 in soybean was especially large, with fewer than half the number of T-RFs at P8 than at any other P concentration, at all the sampling times. Although the mean number of T-RFs was not significantly reduced by high soil P in *Viola*, there was a trend for fewer T-RFs at P8, the probability level for a significant effect was marginal and a *t*-test comparing P1 and P8 does result in a significant difference (*P* < 0.05, data not shown).

Further evidence to show that the AM community at the site was P-responsive comes from the T-RF community analysis. Soybean roots at P8 contained a significantly different T-RF community from the other P concentrations, as did the AM community at P8 in *Viola* (Figs 4 and 5 respectively). In maize, the T-RF community at both P4 and P8 was significantly different from those at P1 and P2. The contrast between the results for colonization and AM community size and structure suggests a degree of compensation, at least in soybean and *Viola*. Although the AM community diversity was reduced, colonization was impacted to a lesser degree. AM fungi that were eliminated or reduced by high soil P were evidently replaced to some extent by AM that were less sensitive to high soil P. This could reflect the persistence of fungi which are able to provide the plant with an extremely economical supply of P, requiring little receipt of C in return, or fungi which receive C from the host to perform functions unrelated to P supply, such as uptake of water or nutrients such as inorganic nitrogen, or even ‘cheaters’, parasites that receive C without providing useful benefits to the host.

In contrast to the influence of soil P, sampling time had a significant influence on AM colonization in all host species, with increased colonization between the first and second sampling time points across soil P concentrations, but a nonsignificant influence on AM diversity. This is consistent with Vandenkoornhuyse *et al*. (2002) and Santos-Gonzalez *et al*. (2007), who showed little change in diversity between different sampling dates, although these results were from perennial grassland species. Daniell *et al*. (2001) showed strong seasonal changes in AM diversity in annual crop species, but the direction of change was inconsistent and sample numbers were small, making interpretation uncertain. In annual plant species, there must be an initial period of colonization by AM fungi, during which there is an increase in diversity, but our results suggest this period is short, and after the initial colonization, overall diversity remains fairly consistent. Changes in the occurrence of T-RFs between sampling times that did occur included both the appearance and disappearance of T-RFs and were confined to rare T-RFs, resulting in nonsignificant differences between communities at different sampling times in NMDS plots. A similar small but insignificant shift in AM communities was recorded by Beauregard *et al*. (2010) in alfalfa, between June and September, while Bainard *et al*. (2012), using a semiquantitative approach to T-RFLP analysis of AM communities in maize, showed a change in abundance of different phylotypes between June and August, but very little change in occurrence. This does not suggest that succession of AM species during the growing season happens to any significant extent once initial establishment has occurred and this hypothesis is supported by results from Mummey *et al*. (2009), which showed that plants preinoculated with *Glomus* spp. retained a similar AM community after transplanting into a field situation. The functional significance of changes in colonization is uncertain. Highest host P demand will be during grain/seed filling, which coincided with the highest degree of colonization in all species, but function is not directly related to the degree of colonization (Smith & Smith, 2011).

Although soil P concentration had a significant influence on the T-RF communities in all the three host plants, the greater influence was host plant identity itself. Only at the highest soil P concentration, when diversity was greatly reduced in all host plants, did soil P override host identity in determining the T-RF communities present. Evidence for host specificity or at least host preference in AM fungi has accumulated over recent years (Sykorova *et al*., 2007a,2007b; Opik *et al*., 2009; Uibopuu *et al*., 2009; Li *et al*., 2010; Hazard *et al*., 2012), overturning the previous paradigm that AM fungi were generalists showing little host preference (Smith & Read, 1997). Li *et al*. (2010) found distinctly different AM communities in three plant species, but in contrast to our results found that habitat was a stronger influence than host plant; however, they gave little information about the two habitats. It is clear that host plant species has a strong influence on the AM community present in roots, but it appears that environmental factors, such as soil P, override this effect if they are large enough, a result also hinted at in Bainard *et al*. (2012). Host plant neighbour identity can also influence the AM community present in roots (Hausmann & Hawkes, 2009, 2010), which may explain the wider range of AM communities present in *Viola* compared with maize and soybean (Fig. 2), *Viola* potentially having a range of neighbour species in the mixed weed flora. This mixed neighbour effect may have been further enhanced or suppressed depending on whether *Viola* germinated before or after its immediate neighbours (Hausmann & Hawkes, 2010).

The significance of the differences in AM community between host plants and the reduction in diversity at high soil P is uncertain. Functional diversity within the AM fungi is a well established fact, with evidence that phylogenetically distantly related AM species are functionally dissimilar (Maherali & Klironomos, 2007). Evidence from our cloning data hints that differences in the T-RF communities in the host plants have at least some basis in phylogeny, suggesting a functional basis. Opik *et al*. (2009) reported distinct AM fungal communities in forest specialist plant species vs generalists and tentatively suggested a functional basis to the difference. Our three host plants, a C3 herb (*Viola*), a C_4_ grass (Maize) and a legume (Soybean), are ecologically distinct and thus would be expected to host distinct communities of functionally different AM fungi if host preference has a functional basis.

Whatever the immediate impact of the reduction in the diversity of AM fungi caused by high soil P on plant growth or nutrient uptake, a significant impact on the AM community in the soil would occur as propagule numbers of the absent species rapidly declined (Troeh & Loynachan, 2003). There were some particularly interesting patterns when individual T-RFs were considered. For instance, all the T-RFs that were found at lower P concentrations and that were unique to soybean were absent at P8, although this was not a pattern repeated with T-RFs unique to *Viola*. This strongly suggests that response to soil P may be dependent on AM identity, rather than random elimination of AM in response to high soil P. Helgason *et al*. (2007) reported a similar effect with a differential impact of disturbance on rare and common AM fungi, in their case in response to application of benomyl, while comparisons between AM communities in agroecosystems managed in different ways often show that AM families, such a Gigasporaceae and Acaulosporaceae, are more easily reduced or eliminated by unsympathetic management than the Glomeraceae, with agricultural ecosystems often dominated by *Glomus* group A (Franke-Snyder *et al*., 2001; Oehl *et al*., 2003; Hijri *et al*., 2006). This may help to explain some of the contradictory results in the literature regarding the influence of soil P on AM fungi. If some AM fungi have already been suppressed because soil P is elevated for historical reasons, then adding P to the soil will have little impact, as the remaining AM fungi are insensitive. The available soil P concentration at P8 (201–280 mg kg^−1^) used in this study was unusually high even for agroecosystems; the AM fungal community diversity in soybean and *Viola* was resistant to moderately high soil P, perhaps because it was very efficient at delivering P to the host, for a small C cost, only being reduced at very high soil P concentrations. The P4 concentration of 46–70 mg kg^−1^, to which AM diversity in maize responded, is, however, well within the range that could be expected in agroecosystems, also indicating a differential response, dependent on host species, possibly reflecting the different ‘value’ placed on P delivered by the AM pathway by different plant species.

The introduction of molecular methods to the study of AM fungi has revealed a previously unsuspected degree of complexity in the ecology of the fungi and in their relationship with the host plant. Here we have provided further evidence of strong host specificity in the AM fungi and evidence that different AM communities colonizing different hosts respond in a broadly similar way to increased soil P. However, we have also shown that there are subtle differences between the responses of different AM communities that may help explain seemingly contradictory results in the literature.

## Supporting Information

Fig S1 Phylogenetic tree showing relationship of sequences from maize and soybean to described species.

Notes S1 Clones sequenced from maize and soybean.
