# Supplementary figures and images for: Contrasting arbuscular mycorrhizal communities colonizing different host plants show a similar response to a soil phosphorus concentration gradient
Source: New Phytol. 2013 Feb 20;198(2):546–56. doi: 10.1111/nph.12169 (PMC3798118; doi:10.1111/nph.12169)

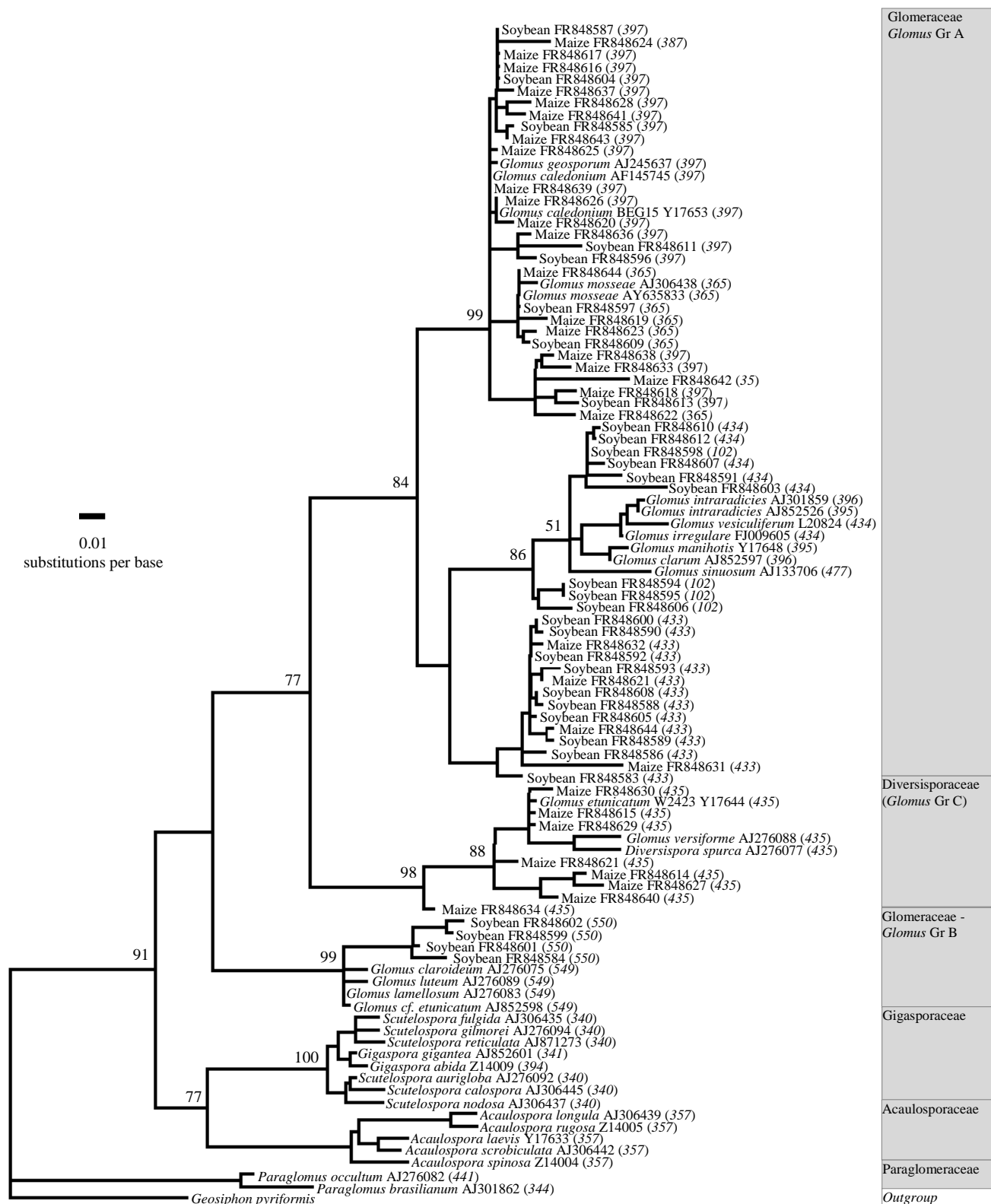

Supplement: Fig S1 — Phylogenetic tree showing relationship of sequences from maize and soybean to described species. [file nph0198-0546-sd1.pdf]
