# Supplementary material for: Contrasting arbuscular mycorrhizal communities colonizing different host plants show a similar response to a soil phosphorus concentration gradient
Source: New Phytol. 2013 Feb 20;198(2):546–56. doi: 10.1111/nph.12169 (PMC3798118; doi:10.1111/nph.12169)
Supplement: Notes S1 — Clones sequenced from maize and soybean. [file nph0198-0546-sd2.docx]

**Supporting Information Notes S1 & Legend to Fig. S1**

**Notes S1**

A total of 87 clones were sequenced from maize and 78 from soybean. There were no contaminant sequences either from maize or soybean. Phylogenetic analysis of non identical clones along with sequences of described AM fungal species produced a topology in close agreement with that published by Schwarzott *et al.,* (2001) and others published in more recent work e.g. Santos *et al,.* (2006), Öpik *et al.,* (2009) (Fig. S1). Seventy five percent of the sequences isolated from maize clustered with described sequences in the putative family *Glomus* group A, (Schwarzott *et al.,* 2001) with 25% clustering with *Glomus* group B, although similarity either to sequences of described species or environmental database sequences was as low as 95% in some cases. Of the sequences isolated from soybean 95% clustered with *Glomus* group A and 5% with group C (Diversisporaceae), with no sequences clustering in *Glomus* group B, though actual similarity to sequences of named species or environmental database sequences was as low as 97%. It is evident from this that the 18S rDNA fragment amplified by this primer set provides poor discrimination between *Glomus* species, which combined with the small inherent uncertainty in T-RF sizing associated with T-RFLP makes it impossible to directly associated clones with named sequences (Fig. S1). Other enzyme label combinations did not give greater discrimination (Data not shown).

**Fig. S1** (Legend; see pdf file for the figure) Phylogenetic tree showing relationship of sequences from maize and soybean to described species. Tree is based on approximately 800 characters in the 18S rDNA region, bootstrapped with 1000 replications, using the neighbour joining method. Distance relationships were obtained using the Kimura-2-Parameter model, with a Gamma distance correction of 0.5 used. Numbers after described species names and clone identifiers refer to accession numbers in EMBL database. Numbers above branches refer to percent bootstrap values. Numbers in brackets refer to Hex labelled T-RFLP fragment size from the HpyCH4III digest used in analyses.
